# Development and comparison of UPLC-ESI-MS and RP-HPLC-VWD methods for determining microcystin-LR

**DOI:** 10.1039/d1ra03521e

**Published:** 2021-06-29

**Authors:** Peng Jin, Kai Yang, Ruining Bai, Mei Chen, Shilin Yang, Kebo Fu, Jieli He

**Affiliations:** College of Pharmacy, Dali University Dali 671000 Yunnan P. R. China hejieli@dali.edu.cn +86-872-2257414; Public Security Bureau Dali Bai Autonomous Prefecture Dali 671000 Yunnan P. R. China

## Abstract

Microcystin-LR (MC-LR) generated by cyanobacteria is a kind of potent hepatotoxin, which poses a considerable threat to human health. In the research field of MC-LR removal, the quantitative analysis in a wide concentration range of samples is inevitable. In this paper, we presented the pseudo united use of an Ultra Performance Liquid Chromatography Mass Spectrometry (UPLC-MS) and High Performance Liquid Chromatography system with a Variable Wavelength Ultraviolet Detector (HPLC-VWD) approach to detect MC-LR. The UPLC-MS system was applied to determine MC-LR in trace concentration because of its high sensitivity. However, it is generally believed that the determination of high concentration samples by UPLC-MS will cause problems such as inaccurate quantification and contamination of ion sources. In consequence, the HPLC-VWD was employed to determine the high concentration of MC-LR. The sensitivity, precision and accuracy of the two methods were compared in detail. The linear ranges of UPLC-MS and HPLC-VWD methods were from 0.08 to 10 μg L^−1^ and 1 to 5000 μg L^−1^, respectively. The detection and quantification limits of UPLC-MS were 0.03–0.05 μg L^−1^ and 0.08 μg L^−1^, and the corresponding two values of HPLC-VWD were 0.6 and 1.0 μg L^−1^. The recoveries of UPLC-MS and HPLC-VWD were 88.5–106.7% and 98.7–101.6%, with the relative standard deviations of 3.72–5.45% and 0.38–1.69%, respectively. The potential adsorption properties of MC-LR on filter membranes with diverse materials and pore sizes were evaluated and the negative results were obtained. The detection of MC-LR by UPLC-MS was free from matrix effects. The presented UPLC-MS and HPLC-VWD methods were used to analyze the water samples from Erhai Lake, which is located in Dali, Yunnan, China. The results of UPLC-MS analysis indicated that the MC-LR was only identified in water samples of Shuanglang Bay and Xier River, with concentrations of 0.120 and 0.303 μg L^−1^, whereas MC-LR was not detected by HPLC-VWD.

## Abstract 2

UPLC-MS and HPLC-VWD methods were used together for MC-LR determination in a wide concentration range. UPLC-MS can be applied in trace MC-LR determination, whereas HPLC-VWD is more suitable for high concentration range detection.

## Graphical abstract

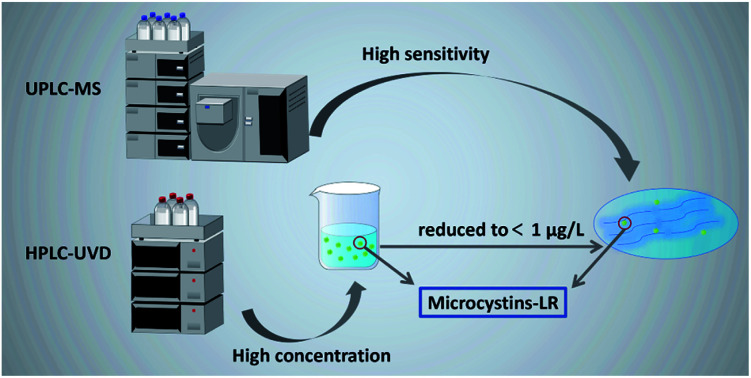

## 1. Introduction

**Fig. 1:**
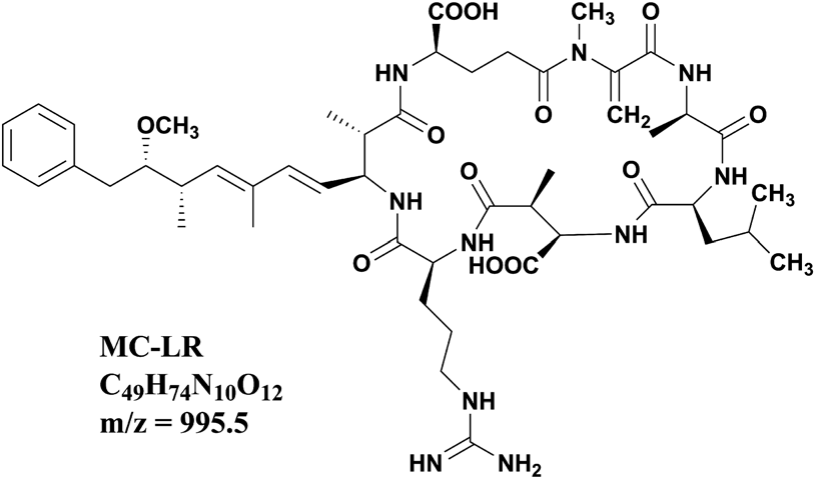
Chemical structural formula of MC-LR.

Nowadays, due to the accelerated eutrophication process of the freshwater body, periodic outbreaks of cyanobacterial blooms have expanded into a global problem. Algal cells release a variety of noxious secondary metabolites called cyanotoxins during growth and after death, and the microcystins (MCs) are the most abundant and common cyanotoxins discharged into aquatic water bodies.^1^ MCs are cyclic heptapeptides with the basic cyclo structure of d-Ala-X-d-*erythro*-β-Methyl Aspartic acid (MeAsp)-Y-(2*S*,3*S*,8*S*,9*S*)-3-amino-9-methoxy-2,6,8-trimethyl-10-phenyldeca-4,6-dienoic acid (Adda)-d-Glu-*N*-methyldehydroalanine (Mdha), and *X*, *Y* are variable l-amino acids.^2^ Microcystin-LR (MC-LR) with leucine (L) and arginine (R) in the positions of *X* and *Y* (Fig. 1) is the most toxic and frequent variant among the over 200 microcystins identified to date.^3^ Many research studies indicated that MC-LR has hepatic toxicity and a tumor promotion effect. Aquatic species, farm animals and human beings would be harmed by MC-LR through exposure *via* drinking, environmental, and recreational waters.^4–8^ The International Agency for Research on cancer considered MC-LR to be a category 2B carcinogen. Therefore, the removal of MC-LR has attracted extensive attention in recent years, and several technologies have been exploited including adsorption, photocatalytic degradation, Fenton oxidation, electrochemical oxidation and biodegradation.^9–12^ The removal rate of MC-LR is one of the most important indexes to evaluate these techniques. Thus, accurate quantitative and sensitive determination of MC-LR concentration during the removal process is truly essential. In most research systems of MC-LR removal, the initial concentrations of MC-LR are usually as high as 5 mg L^−1^ or even higher.^13–19^ After effective removal, MC-LR concentrations are lower than 1.0 μg L^−1^ to meet the requirement of the WHO guidelines. How to accurately monitor MC-LR in such a wide concentration range has always been a thorny problem.

To date, several analytical techniques have been employed to determine MC-LR, such as enzyme-linked immunosorbent assays (ELISA),^20–23^ protein phosphatase inhibition assays (PPIA),^24,25^ chemical sensors and biosensors,^26–32^ high performance liquid chromatography (HPLC)^33,34^ and high performance liquid chromatography mass spectrometry (HPLC-MS).^35–38^ ELISA is one of the most common and sensitive bioassay techniques for MC-LR detection. However, there are many interference factors in the process of ELISA test, which may produce false-positive results. PPIA consume expensive reagents and special operation circumstance. Most sensor detection systems have a limited test frequency. Among these methods, HPLC coupled with a variable wavelength ultraviolet detector (VWD) is the most widely used technology.^39^ At present, the majority MC-LR removal systems employ HPLC-VWD for MC-LR determination. Nevertheless, the sensitivity of VWD is low and additional clean-up and concentration steps are commonly needed.^39^ Up to now, solid phase extraction (SPE) is typically adopted to extract and concentrate the samples in aqueous solution, and HPLC-VWD after SPE pretreatment for MCs determination in water is listed as China National Standard Method (GB/T 20466-2006). And yet SPE process is comparatively expensive because of the large amounts of organic solvent using. Over the years, solid phase microextraction (SPME) technique is developed with the advantages of no use of organic solvent and no need of cleaning up procedure.^40,41^ However, SPME fibers are usually costly and have limited lifetimes. Anyhow, the necessary sample preparation procedures are time-consuming when MC-LR concentration is very low. Compared with HPLC-VWD, ultra performance liquid chromatography tandem mass spectrometry (UPLC-MS) can get higher sensitivity and selectivity.^42^ Therefore, UPLC-MS is quite suitable for low concentration range. Nevertheless, it is generally recognized that the high concentration samples will possibly pollute the ion source of mass spectrometer and lead to serious column residue. In comparison, HPLC columns are less likely to cause column residues.

Here, to help choose the proper analysis method in wide concentration range of MC-LR, UPLC-MS and HPLC-VWD methods were proposed, and the sensitivity, accuracy and precision of the two methods were compared in detail. The suitable concentration ranges of the two methods were evaluated. Moreover, the proposed analysis methods of UPLC-MS and HPLC-VWD were successfully applied to detect MC-LR in surface water samples.

## 2. Experimental

### 2.1 Reagents

The MC-LR standard solution was obtained from Express Technology Co., Ltd. (Beijing, China), with a purity of over 98%. All reagents were chromatographic grade. Methanol and formic acid were respectively purchased from Honeywell International Company (Charlotte, USA) and Thermo Fisher Scientific (Massachusetts, USA). Acetonitrile and trifluoroacetic acid were obtained from Aladdin Industrial Corporation (Shanghai, China). The dilution water used was ultrapure water produced by a water purification system (DZG-303A, Tangle Corning Technology Factory, China). Filter membranes [nylon (NY), mixed cellulose (MCE), glass fiber (GF), polytetrafluoroethylene (PTFE), polyethylene sulfoxide (PES)] with a diameter of 13 mm and pore sizes of 0.22 and 0.45 μm were purchased from Tianjin Jinrong Experimental Equipment Company (Tianjin, China).

The MC-LR stock solution was prepared at the concentration of 10 mg L^−1^ in methanol and stored at −20 °C. A series of MC-LR standard working solutions ranging from 0.08 to 5000 μg L^−1^ were prepared in 20% methanol for the preparation of calibration curves and for spiking samples. The surface water samples were filtered for three times by PES filter membranes with a diameter of 13 mm and a pore size of 0.22 μm and then stored at 4 °C.

### 2.2 UPLC-MS instrumentation

Qualitative and quantitative analysis of MC-LR was performed on an Ultimate 3000 HPLC system coupled with a triple quadrupole mass spectrometer (Ultimate 3000-TSQ Quantis) fitted with an electrospray ionization source (ESI). A hypersil gold column (1.9 μm particle size, 100 mm × 2.1 mm i.d.) thermostated at 30 °C was used for separation. The gradient mobile phase was consisted of 0.05% formic acid in water as solvent A and methanol as solvent B. The flow rate was 0.40 mL min^−1^. The optimized gradient elution program was as follows: 0–1.5 min, 10–90% solvent B; 1.5–3 min, 90% solvent B; 3–3.01 min, 90–10% solvent B; 3.01–6 min, 10% solvent B. ESI was working in positive ion electrospray ionization mode with selective reaction monitoring (SRM) for MC-LR quantification. The monitoring parameters were optimized as follows: ion source spray voltage, 3000 V; sheath gas flow rate, 45 L min^−1^; auxiliary gas flow rate, 15 L min^−1^; ion transfer tube temperature, 350 °C; atomization temperature, 300 °C.

### 2.3 HPLC-VWD instrumentation

HPLC was coupled with a variable wavelength UV detector, and a reverse-phase (RP) Agilent XDB-C18 column (250 mm × 4.6 mm i.d., 5 μm particle size) was used. The column temperature and flow rate were maintained at 35 °C and 1 mL min^−1^, respectively. The injection volume was 25 μL, and the detection wavelength was fixed at 238 nm. The equipped UV detector has a response time of 0.063 s and an attenuation factor of 125 mAU. The mobile phase with a gradient elution system is composed of 0.05% trifluoroacetate in water (A) and acetonitrile (B). The gradient conditions of mobile phase were 0 min 80% A, 4–4.5 min 10% A and 6–15 min 80% A.

### 2.4 Methods validation

#### 2.4.1 Calibration curves and linearity

Calibration curves of UPLC-MS were obtained by analyzing standard solutions at seven concentration levels between 0.08 and 10 μg L^−1^. Calibration curves of HPLC-VWD were obtained by analyzing standard solutions at seven concentration levels between 0.001 and 5 mg L^−1^. The calibration curves were constructed by plotting the peak area *versus* the seven levels concentrations of MC-LR with linear regression. Linearity was considered satisfactory if the *R*^2^ value was higher than 0.99.

#### 2.4.2 Accuracy and precision

Method accuracy was evaluated by a blank spiked recovery experiment (in sextuplicate). The accuracy of UPLC-MS was evaluated in blank surface water spiked at three concentration levels (0.1, 0.2, and 1 μg L^−1^). To evaluate the accuracy of HPLC-VWD, 20% methanol–water was used as blank sample spiked at 0.2, 0.5, and 1.0 mg L^−1^. Accuracy was assumed satisfactory if recoveries were in the range of 80–120%. Method precision was assessed by repeated experiments. Three different levels of concentration (0.1, 1.0 and 10 μg L^−1^) and (1.0, 10 and 1000 μg L^−1^) were prepared to examine the repeatability of the UPLC-MS and HPLC-MS, respectively. All precision experiments were measured in parallel for 6 times under the above instrument conditions.

## 3. Results and discussion

### 3.1 Confirmation of MS signals

**Fig. 2:**
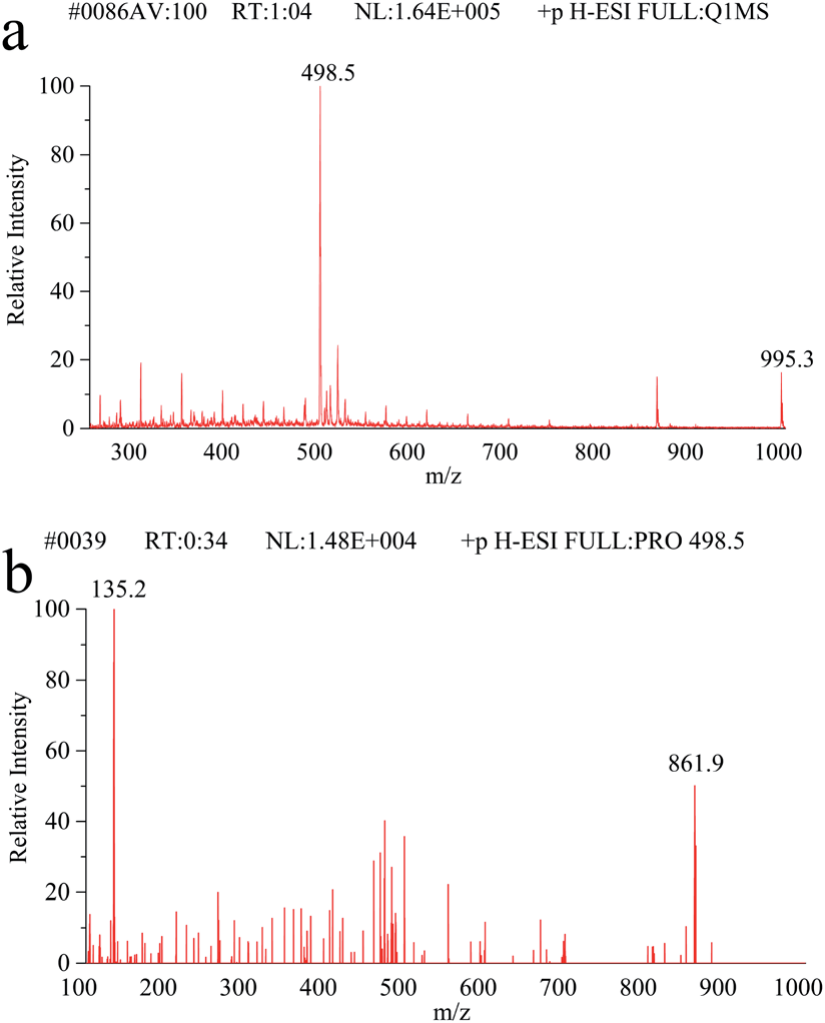
(a) The full scan mass spectrum of MC-LR. (b) The fragment scan mass spectrum.

**Fig. 3:**
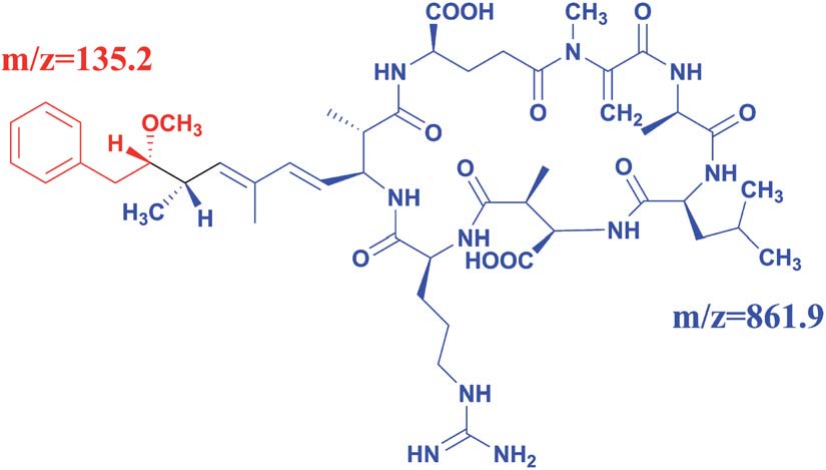
Chemical structure of MC-LR corresponding to *m*/*z* = 135.2 and *m*/*z* = 861.9.

Full scan mass spectra and product ion scan mass spectra of MC-LR (5 mg L^−1^) were obtained by mass spectrometry without chromatography column separation with the scan range from *m*/*z* 100 to 1000. In the positive ion mode, the single charged protonated molecular ion ([M + H]^+^) at *m*/*z* 996.1 was supposed preferentially selected as the precursor ion. However, after optimization of MS parameters, we found that the precursor ion of [M + H]^+^ was not the most abundant. As shown in the full scan spectrum (Fig. 2a), the double charged ion [M + 2H]^2+^ at *m*/*z* 498.5 was the dominant ion and thus selected as the precursor ion. It is probably because MC-LR is a cyclic heptapeptide compound, which is prone to form multi-charged ions [M + *n*H]^*n*+^ instead of [M + H]^+^ in the electrospray ion source.^42^ With the different relative collision energies applied to the precursor ion, MC-LR will be dissociated into fragments along its characteristic pathway. As shown in Fig. 2b, two foremost product ions (*m*/*z* 135.2 and 861.9) appeared. The fragment ions at *m*/*z* 135.2 and 861.9 were attributed to the fragment [PheCH_2_CHOCH_3_]^+^ of the Adda moiety and parent molecular ion after loss of 135.2 mass unit (Fig. 3), respectively. The product ion (*m*/*z* = 861.9) with higher charge mass ratio was selected as the qualitative ion, and the most abundant product ion at *m*/*z* 135.2 was set as the quantitative ion. The mass spectrometry parameters including retention time, molecular mass, collision energy, precursor ions and fragment ions are summarized in Table 1.

**Table 1:** Analytical parameters of UPLC-MS analysis of MC-LR

Compound name	Precursor ion (*m*/*z*)	Product ion (*m*/*z*)	Collision energy (V)	Retention times (ms)
MC-LR	498.5	135.2	56.2	248.3
		861.9	45.1	

### 3.2 Evaluation of filter membranes

**Fig. 4:**
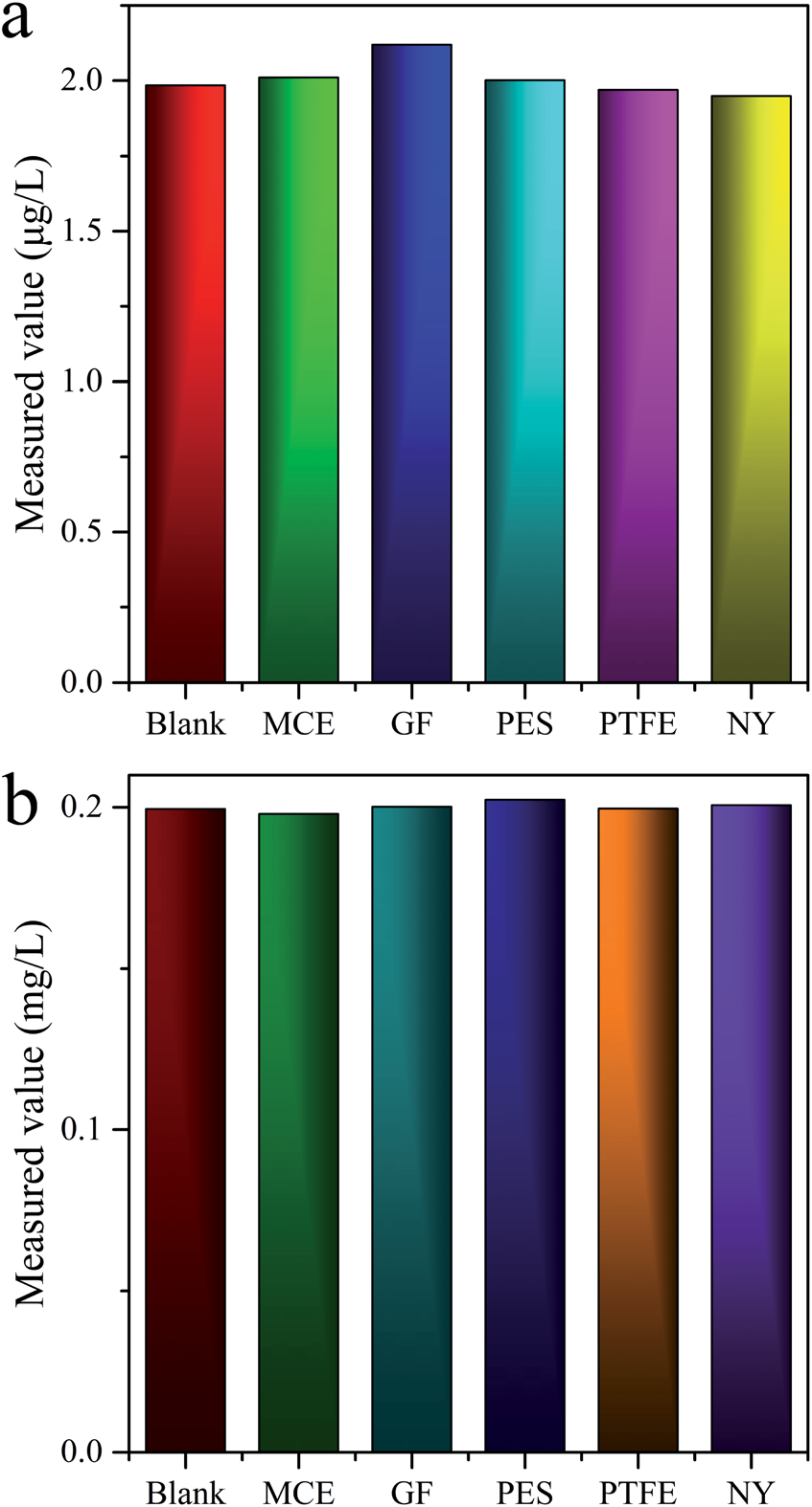
Adsorption effects of filter membranes evaluated by (a) UPLC-MS and (b) HPLC-VWD analysis.

There was reported in the literature that the filter membranes would adsorb MC-LR in the pretreatment process, which lead to inaccurate quantitative results.^43^ For purpose of investigating the adsorption behaviors of MC-LR on filter membranes, five filter membranes consisted of different materials [nylon (NY), mixed cellulose (MCE), glass fiber (GF), PTFE, PES] were adopted. The standard solutions of 2 and 200 μg L^−1^ MC-LR were filtered by above membranes with pore sizes of 0.22 and 0.45 μm and then detected by UPLC-MS and HPLC-VWD, correspondingly. The evaluation results of UPLC-MS (Fig. 4a) and HPLC-VWD (Fig. 4b) showed that there were no significant differences in the measured values between the filtered and unfiltered samples, indicating the adsorption effects of all tested filter membranes can be ignored. The PES filter membrane with pore size of 0.22 μm was used in the following experimental studies for both UPLC-MS and HPLC-VWD methods.

### 3.3 Matrix effects

Matrix effects have always been a vexing problem in LC-MS analysis. Endogenous and exogenous impurities including inorganic salts and other matrix components are often present in electrospray analysis, which can seriously affect the ionization process of the target compounds. To evaluate the influence of possible matrix effects on mass spectrometry analysis, two 2 μg L^−1^ MC-LR solutions were prepared, one using 20% methanol–ultrapure water solution and the other using 20% methanol–surface water solution as solvents, respectively. The two MC-LR solutions were analyzed for 6 parallel measurements. As shown in Table 2, the average measured values of matrix solution and standard solution were 1.94 and 1.92 μg L^−1^. The value of matrix effect calculated by relative response of the two solutions was 101%, indicating the matrix effect had almost no influence on the method. We considered that in our proposed analysis system, the pre-treatment process is quite facile, which commonly will not introduce foreign impurities. Moreover, the concentrations of organic and inorganic substances in water are usually quite low.

**Table 2:** The evaluation of matrix effect^a^

Solvent	Measured value (μg L^−1^)	Average values (μg L^−1^)	Average recovery (%)	RSD (%, *n* = 6)
20% methanol–ultrapure water	1.86	1.92	96.20	3.46
	2.22			
	1.86			
	2.08			
	1.74			
	1.78			
20% methanol–surface water	2.12	1.94	96.95	8.55
	2.12			
	1.79			
	1.80			
	1.79			
	2.02			

a Recovery (%) = (measured value/spiked value) × 100%.

### 3.4 Methods validation

The sensitivity, precision, accuracy and linear range of UPLC-MS and HPLC-VWD were compared by validation of the two analytical methods.

#### 3.4.1 Calibration and linearity

**Fig. 5:**
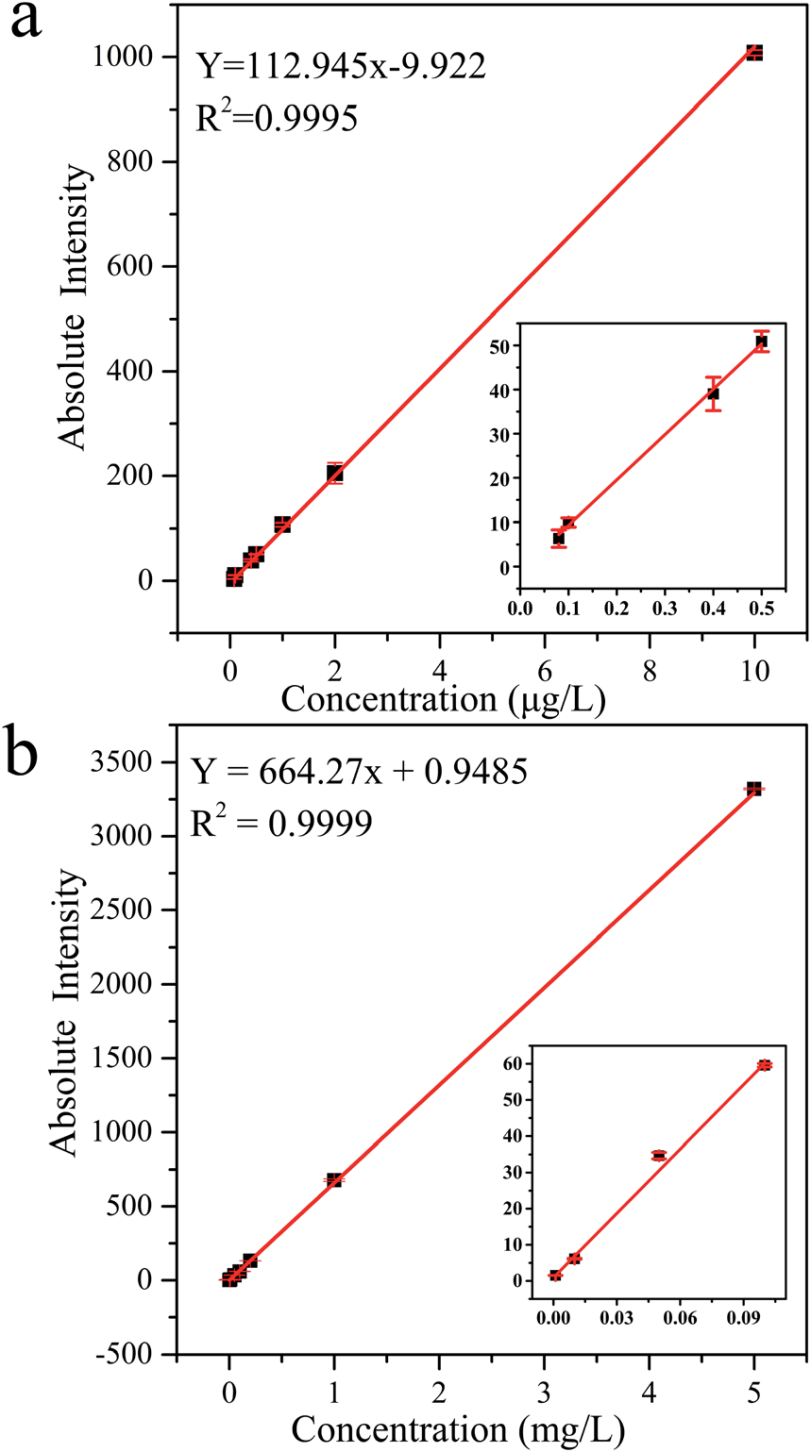
Calibration curves and regression equations of MC-LR standard solutions tested by (a) UPLC-MS and (b) HPLC-VWD methods.

Fig. 5 shows the calibration curves of MC-LR standard solutions determined by UPLC-MS and HPLC-VWD techniques. The correlation coefficients were both greater than 0.999, indicating that the concentrations of MC-LR were well correlated within the linear range. The linear range of UPLC-MS, from 0.08 to 10 μg L^−1^, was narrower than that of HPLC-VWD, which ranged from 1 to 5000 μg L^−1^. Three times of signal/noise ratio (S/N) was calculated as the limit of detection (LOD). In this way, the LOD values of MC-LR were 0.03–0.05 μg L^−1^ for UPLC-MS and 0.6 μg L^−1^ for HPLC-VWD. The limit of quantification (LOQ) values calculated by 10 times S/N were 0.08 and 1 μg L^−1^ for UPLC-MS and HPLC-VWD methods, respectively. The above results indicated that UPLC-MS method exhibited much greater sensitivity, and HPLC-VWD technique had wider linear range. The LOQ of MC-LR determined by UPLC-MS was below the WHO guideline of 1 μg L^−1^ in drinking water. Therefore, the proposed UPLC-MS could meet the demand of sensitivity in MC-LR survey.

#### 3.4.2 Accuracy and precision

Table 3 shows the recovery and relative standard deviation (RSD) of MC-LR detected for six parallel tests by the two analytical methods. Accuracy is usually expressed as the recovery of known and added amounts of analyte. The recovery was calculated as measured value/spiked value × 100%. As displayed in Table 3, the average recoveries of MC-LR tested by UPLC-MS and HPLC-VWD at the three fortification levels assay were determined as 88.5–106.7% and 98.7–101.6%, respectively, indicating the accuracies of the two methods were quite satisfactory. Precision reflects the analysis deviation and is expressed as the RSD. The RSD of HPLC-VWD method in the concentration range of 200–1000 μg L^−1^ was 0.38–1.69%, which was lower than that of UPLC-MS with the value of 3.72–5.45% in the concentration range of 0.1–1 μg L^−1^. The results suggested that the HPLC-VWD method has better precision in the proper high concentration of MC-LR, which is reasonable.

**Table 3:** UPLC-MS and HPLC-VWD recovery experiments^a^

Solvent	Measured value (μg L^−1^)	Average values (μg L^−1^)	Average recovery (%)	RSD (%, *n* = 6)
20% methanol–ultrapure water	1.86	1.92	96.20	3.46
	2.22			
	1.86			
	2.08			
	1.74			
	1.78			
20% methanol–surface water	2.12	1.94	96.95	8.55
	2.12			
	1.79			
	1.80			
	1.79			
	2.02			

a Recovery (%) = (measured value/spiked value) × 100%.

**Fig. 6:**
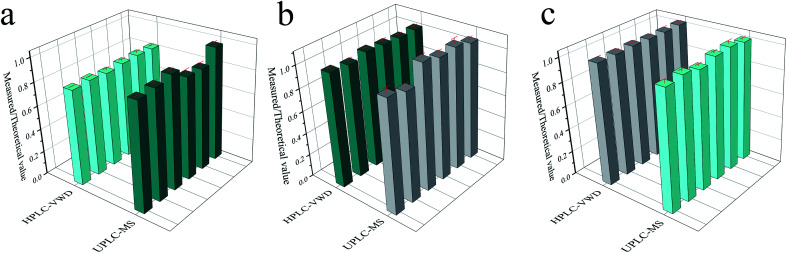
The ratio of measured to theoretical value of MC-LR analyzed by HPLC-VWD and UPLC-MS at the concentrations of 1 and 0.1 μg L^−1^ (a), 10 and 1 μg L^−1^ (b), 1000 and 10 μg L^−1^ (c), respectively. (b) and (c) Comparison of precision between UPLC-MS and HPLC-VWD. Relative intensity is the ratio of measured to theoretical values.

In order to compare the accuracy and precision of the two methods in a wider concentration range, the ratios of the measured results (parallel 6 times) to the theoretical values were calculated and displayed in Fig. 6. The RSD values were 1.19%, 1.25% and 0.22% for HPLC-VWD analysis at the concentrations of 1, 10 and 1000 μg L^−1^, respectively. For UPLC-MS detection, the RSDs were 4.99%, 5.26% and 1.55% at the concentrations of 0.1, 1 and 10 μg L^−1^, correspondingly. It can be concluded that the HPLC-VWD method exhibited higher precision in such a wide concentration range. In addition, the mean ratios were 82%, 104% and 102% at the concentration of 1, 10 and 1000 μg L^−1^ for HPLC-VWD, and 91%, 104% and 101% at 0.1, 1 and 10 μg L^−1^ for UPLC-MS analysis, which indicated that UPLC-MS displayed higher accuracy, especially in trace detection.

### 3.5 Statistic evaluation

The significant difference between UPLC-MS and HPLC-VWD analysis methods was checked by statistic evaluation for testing of MC-LR at ppb (parts per billion) level. MC-LR standard solution of 1 μg L^−1^ was prepared and determined by UPLC-MS and HPLC-VWD respectively (parallel 6 times). The measured values, mean values, standard deviations (*S*) and variances (*S*^2^) were summarized in Table 4. Considering that the outliers may affect the accuracy and precision of the results, the Grubbs test was used to determine whether outliers should be discarded. A 95% confidence level was selected in the statistical evaluation. The *G* value was calculated by formula (1), where *x*_q_ is questionable value and *x̄* is average value. The critical value of *G* is *G*_0.05, 6_ = 1.89.^44^1
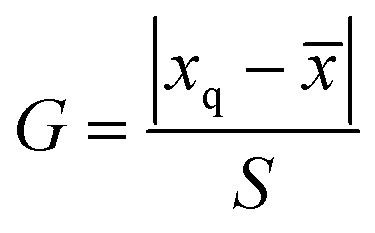
The questionable value of UPLC-MS analysis was 0.9558, by calculating the *G* value was 1.5045 and lower than *G*_0.05, 6_, so the *x*_q_ should be retained. For HPLC-VWD measurement, the *x*_q_ = 0.8754, the calculated *G* = 1.5869, which was also lower than *G*_0.05, 6_, the *x*_q_ of 0.8754 also should be retained. After checking the outliers, *F* test was used to determine whether there was a significant difference in the precision of the two sets of data obtained by the UPLC-MS and HPLC-VWD methods. The *F* value was calculated by formula (2).2
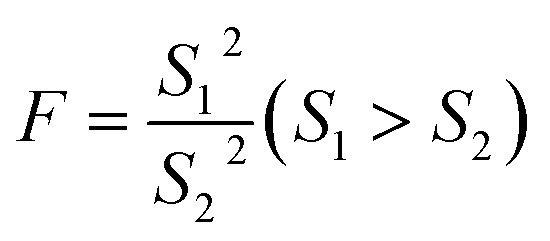
*S*12 and *S*22 were the variances of the two sets of data. According to regulation, the large variance is the numerator and the small one is the denominator. Compare the calculated *F* value with the one-sided critical value *F*_*α*, *f*_1_, *f*_2__ of the variance ratio. If *F* < *F*_*α*, *f*_1_, *f*_2__, it means that there is no significant difference in the precision of the two sets of data, which would otherwise mean a significant difference. Among them, *f*_1_ and *f*_2_ are the degrees of freedom of the two sets of data (*f* = *n* − 1), and the value of *F*_0.5, 5, 5_ is 5.05. It was calculated that *F* = 1.5328, which was smaller than *F*_0.5, 5, 5_, indicating that there was no significant difference in the precision of the two methods. After that, the *t* test was used to evaluate the systematic error of the two sets of data. Formulas (3) and (4) were used to calculate *t* and *S*_R_ (pooled standard deviation). *x̄*_1_ and *x̄*_2_ are the mean values of the two sets of data respectively, *n*_1_ and *n*_2_ are the measurement times of the two sets of data respectively, and the *t* critical value is 2.228.^44^3
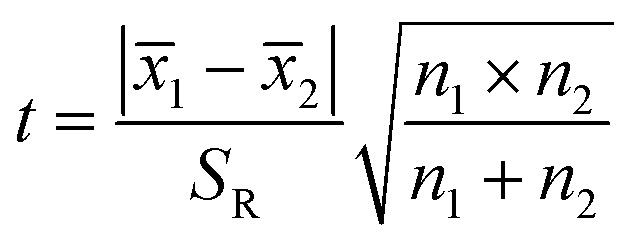
4
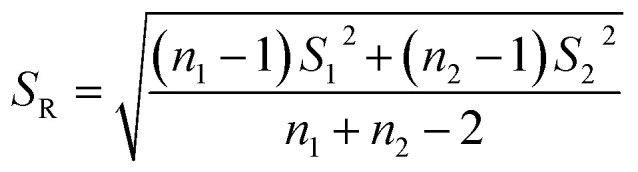
By calculation, *S*_R_ = 0.0499, *t* = 8.1014, and the *t* value was larger than the *t* critical value of the two-sided test (*t*_0.05, 10_ = 2.228), implying there was a significant difference in mean value of the two analysis methods, and the systematic errors of the two methods can not be ignored. Based on the above analysis, for detection of 1 ppb MC-LR, the two methods had the similar precision, but the mean values were significantly different. In addition, the absolute errors between the theoretical values and measured data analyzed by UPLC-MS and HPLC-VWD were 0.0385 and 0.1948 μg L^−1^, implying the HPLC-VWD method was not suitable for the quantification of MC-LR at ppb level.

**Table 4:** Statistic evaluation

Methods	Measured values (μg L^−1^)	Mean values (μg L^−1^)	*S*	*S* ^2^
UPLC-MS	1.1046	1.0385	0.0549	0.0030
	1.0749			
	1.0650			
	1.0352			
	0.9955			
	0.9558			
HPLC-VWD	0.8754	0.8052	0.0443	0.0020
	0.8453			
	0.7851			
	0.7851			
	0.7700			
	0.7700			

### 3.6 Determination of surface water samples

**Fig. 7:**
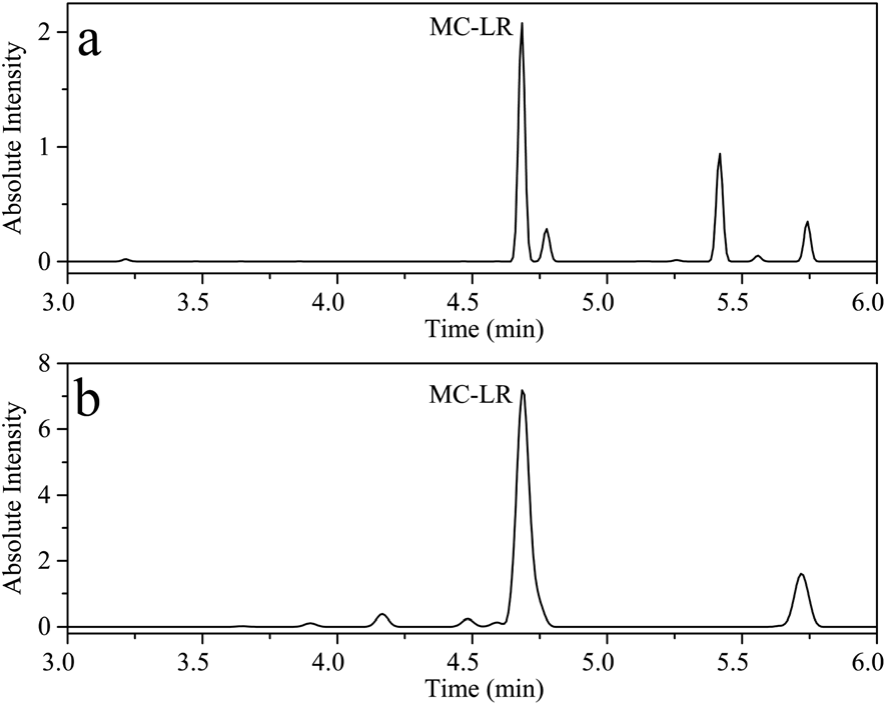
UPLC-MS chromatogram of (a) Shuanglang Bay water sample and (b) Xier River water sample.

Erhai Lake, located in Dali, Yunnan, China, is the second largest fresh water lake in Yunnan Province, with an area of about 252.91 km^2^. Cyanobacteria blooms have occurred several times in history. According to reports, the highest concentration of MC-LR detected at 18 sampling points in Erhai Lake in November 2014 was 0.035 μg L^−1^.^45^ To evaluate the application of the proposed UPLC-MS and HPLC-VWD in surface water samples monitoring, two methods were employed to evaluate MC-LR concentration in Erhai Lake. Nine samples were collected in October 2020 by Dali's Environmental Monitoring Bureau. The sampling sites of the nine water samples were Shuanglang Bay, Haichao Bay, Shacun Bay, Majiuyi Bay, Shaping Bay, Wase Bay, Xiangyang Bay, Hongshan Bay and Xier River, which covered the entire Erhai Lake basin. UPLC-MS studies showed that MC-LR was only detected in Shuanglang Bay and Xier River water samples, with concentrations of 0.120 and 0.303 μg L^−1^, respectively, and the chromatograms were displayed in Fig. 7. However, MC-LR could not be detected by HPLC-VWD in any water sample, because the concentrations of MC-LR were lower than the LOD of HPLC-VWD (0.6 μg L^−1^). Although the maximum concentration of MC-LR in Erhai Lake was within the WHO limit, the protection of ecological environment still required attention.

## 4. Conclusions

Pseudo united use of UPLC-MS and HPLC-VWD methods were developed to meet the quantitative requirements of MC-LR in different research fields. The pre-treatments of the two methods are rather simple and just with filtration before analysis. The adsorption effects of filter membranes and matrix effects can be neglected. Both the two methods have satisfactory reliability, precision and accuracy in their linear ranges. The UPLC-MS has higher sensitivity for MC-LR detection with LOD and LOQ of 0.03–0.05 and 0.08 μg L^−1^, which is efficient to monitor MC-LR in trace concentration, especially under 1.0 μg L^−1^. The proposed HPLC-VWD is more suitable for the high concentration range detection. Thus, UPLC-MS and HPLC-VWD methods should be combined use in MC-LR analysis within a wide range concentration. Moreover, the developed UPLC-MS was successfully applied to survey surface water samples collected in Erhai Lake, which would provide useful data reference for environmental monitoring.

## Conflicts of interest

There are no conflicts to declare.

## Supplementary Material
